# Association of ambient air pollution exposure with psychological distress in mid and later adulthood: A 26-year prospective cohort study

**DOI:** 10.1371/journal.pone.0320332

**Published:** 2025-03-26

**Authors:** Thomas Canning, Marcus Richards, Anna L. Hansell, John Gulliver, Rebecca Hardy, Jorge Arias-de la Torre, Stephani L. Hatch, Ian S. Mudway, Amal R. Khanolkar, Helen L. Fisher, Ioannis Bakolis

**Affiliations:** 1 Department of Biostatistics and Health Informatics, Institute of Psychiatry, Psychology & Neuroscience, King’s College London, London, United Kingdom; 2 Social, Genetic & Developmental Psychiatry Centre, Institute of Psychiatry, Psychology & Neuroscience, King’s College London, London, United Kingdom; 3 Centre for Mental Health Policy and Evaluation, Health Service and Population Research Department, Institute of Psychiatry, Psychology & Neuroscience, King’s College London, London, United Kingdom; 4 MRC Unit for Lifelong Health and Ageing at UCL, University College London, London, United Kingdom; 5 Centre for Environmental Health and Sustainability, University of Leicester, Leicester, United Kingdom; 6 National Institute for Health Research (NIHR) Health Protection Research Unit (HPRU) in Environmental Exposures and Health at the University of Leicester, Leicester, United Kingdom; 7 National Institute for Health Research (NIHR) Leicester Biomedical Research Centre (BRC), Leicester General Hospital, Leicester, United Kingdom; 8 Population Health Research Institute, City St George’s, University of London, London, United Kingdom; 9 Social Research Institute, University College London, London, United Kingdom; 10 School of Sport, Exercise and Health Sciences, Loughborough University, Loughborough, United Kingdom; 11 CIBER Epidemiology and Public Health (CIBERESP), Madrid, Spain; 12 Institute of Biomedicine (IBIOMED), University of Leon, Leon, Spain; 13 Care in Long Term Conditions Research Division, Florence Nightingale Faculty of Nursing, Midwifery & Palliative Care, King’s College London, London, United Kingdom,; 14 Department of Psychological Medicine, Institute of Psychiatry, Psychology & Neuroscience, King’s College London, London, United Kingdom; 15 ESRC Centre for Society and Mental Health, King’s College London, London, United Kingdom; 16 Population Health Improvement UK (PHI-UK), Population Mental Health Consortium, London, United Kingdom; 17 MRC Centre for Environment and Health, School of Public Health, Faculty of Medicine, Imperial College London, London, United Kingdom; 18 NIHR Health Protection Research Units in Environmental Exposures and Health, and Chemical and Radiation Threats and Hazards, Imperial College London, London, United Kingdom; 19 Department of Population Health Sciences, School of Life Course & Population Sciences, King’s College London, London, United Kingdom; The University of Tokyo Graduate School of Medicine Faculty of Medicine: Tokyo Daigaku Daigakuin Igakukei Kenkyuka Igakubu, JAPAN

## Abstract

**Background:**

Existing evidence on associations between exposure to air pollution and psychological distress from middle to older age is limited by consideration of short exposure periods, poor historical covariates, exposures and outcomes, and cross-sectional study designs. We aimed to examine this association over a 26-year period between ages 43 and 69.

**Methods:**

We utilised data from the Medical Research Council National Survey of Health and Development Study (the 1946 British birth cohort). Land-use regression models estimated exposure to specific air pollutants using household addresses for 1991 (NO_2_), 2001 (PM_10_, NO_2_), and 2010 (NO_2_, NO_x,_ PM_10_, PM_2.5_, PM_coarse,_ PM_2.5_abs). These were linked to the closest data collection wave at ages 43, 53 and 60-64, respectively. Psychological distress was assessed through the 28-item version of the General Health Questionnaire (GHQ-28), at ages 53, 60-64 and 69. Associations between each of the pollutants with psychological distress were analysed using generalised linear mixed models, adjusted for pollution exposure before age 43, assigned sex, social class, smoking status, neighbourhood deprivation, and previous mental health problems. We also examined effect modification by social class.

**Results:**

At age 69, 2125 participants completed the GHQ-28. In fully adjusted models, higher NO_2_ exposure was associated with higher GHQ-28 scores across a 26-year period (β=0.023, 95%CI:0.005, 0.040 per interquartile range increase in exposure), whereas higher exposure to PM_10_ was associated with lower GHQ-28 scores across a 16-year period (β=-0.021, 95%CI:-0.037, -0.006). There was no evidence of associations between exposure to other pollutants at age 60-64 and GHQ-28 at age 69. We found no effect modification by social class.

**Conclusions:**

In this cohort there was some evidence of an association between higher cumulative exposure to NO_2_ and higher psychological distress, but mixed associations with other exposures. Policies to reduce pollutant exposure may help improve psychological symptoms in middle to late adulthood.

## Introduction

Outdoor air pollution is one of the leading health challenges worldwide and a key environmental determinant of health [1]. Given the large burden of mental health problems worldwide [2], understanding the extent to which air pollution exposures are associated with poorer mental health is essential for population health.

Psychological distress can be conceptualised as a measure of anxiety, depression, and related symptomology. Such mental health problems can have substantial impacts on middle-aged and older people, limiting autonomy, hindering daily working and family life, and exacerbating other health problems [3]. Prevention of psychological distress in this age group is therefore a key focus for public health policy. Air pollution could be one such target for primary prevention and public health policies as it is a potentially modifiable risk factor [4].

Air pollution is a complex mix of particulate matter (e.g., particulate matter smaller than 2.5μm - PM_2.5_) and gases, such as nitrogen dioxide (NO_2_) and sulphur dioxide (SO_2_). It has established causative associations with physical ill-health and contributes to approximately 36,000 deaths in the United Kingdom each year, and at a cost of $6.43 trillion USD globally [5,6]. These figures reflect adverse impacts on cardiopulmonary conditions, but there is now emerging evidence of suggestive causal links to a range of neurodegenerative conditions and cognition [7]. There is also evidence suggesting that both short and long term exposures to elevated air pollutant concentrations are associated with increased risk of mental health problems [8,9], including incidence of depression [8–11], anxiety [11,12], psychological distress [13,14], co-occurrent long-term conditions [15], with severity of psychiatric disorders [16,17], and within specific age groups, including older adults [12,18–20]. There are also plausible mechanisms for an association, whereby exposure to air pollution can, either through direct transport across the blood brain barrier, or secondary to general systemic inflammation, trigger processes such as neuroinflammation, apoptosis, or neurotransmitter production changes, that may be reflected in psychological distress over time [21].

However, uncertainty remains in understanding the complex, developmental relationship between air pollution and mental health [22,23]. This in part reflects methodological limitations, with air pollution exposure estimates often restricted to relatively short exposure periods (e.g., under 1 year) which may not adequately capture long-term trends in exposures or capture the role of chronic or cumulative exposure in the development of mental health problems across the life course [21,24]. Furthermore, many previous studies have focused on large mixed age cohorts, or on the transition from childhood into adolescence and early adulthood, before age 30, and so often have not evaluated associations in the context of historical mental health problems or exposures [8,13,25]. In this study, we investigate the important transitional period between mid-adulthood (approximately age 40–60) into later adulthood or older age (e.g., age 60+) [26], a point in the life course with limited, but growing, evidence of association between air pollutant exposure and poorer mental health [12,19,20] and where life circumstances often change from employment and family life to retirement and declining physical health. It has been recognised that addressing mental health problems in this age group may help support healthy ageing beyond age 70 [27].

Lastly, there is limited evidence regarding how associations between air pollution exposure and mental health may differ between subgroups within the population. For instance, people with disadvantaged socioeconomic position (SEP) may be at risk of higher exposure to air pollution as well as being more susceptible to adverse health outcomes [28,29]. There has been some limited investigation of this in associations with mental health, with one study [10] finding no evidence of interaction between SEP, based on home ownership, and higher air pollution exposure in relation to common mental health problems. SEP based on occupation, which will be utilised in the current study, may be reflective of general SEP but may also capture differences in workplace-related stressors that could further moderate the pollution-mental health relationship, such as a worsening of physical health or disruption of sleep-wake patterns more commonly found in manual jobs [30,31]. Understanding differences in associations between manual and non-manual occupations may help inform targeting of preventive interventions to those most at risk.

While evidence of an adverse association has increased in quantity and quality in recent years [8,9], there is a lack of studies covering middle to old age that utilise longitudinal study designs with multiple exposure measurements of air pollution and mental health outcomes, while adjusting for early life exposure and prior mental health problems. This study seeks to address this by examining associations between exposure to outdoor air pollution and psychological distress, over a 26-year period between the ages of 43 and 69. We utilised a UK-based birth cohort followed up to 69 years of age with repeated measures of air pollution exposure and psychological distress. First, we examined longitudinal associations between exposure to a range of pollutants from mid-late adulthood (from age 43) and subsequent psychological distress (from age 53). We hypothesised that i) long-term exposures to elevated concentrations of pollutants would be associated with higher psychological distress over a 26-year (NO_2_), 16-year (particulate matter smaller than 10μm (PM_10_)) and 5-year (nitrogen oxides (NO_x_), PM_2.5_, particulate matter 2.5µm to 10µm (PM_coarse_), and particulate matter absorbance as a measure of black carbon absorption fraction (PM_2.5_abs)) period. Secondly, we examined whether social class modified associations between air pollution and psychological distress. We hypothesised that ii) the association between air pollution exposure and psychological distress in mid-late adulthood would be stronger among those in lower socio-economic positions vs. those in higher positions, utilising a proxy of SEP for participants in manual vs. non-manual jobs.

## Methods

### Sample

The Medical Research Council (MRC) National Survey of Health and Development (NSHD) is a sample originally consisting of 5,362 (out of 13,687) singleton births from all births to married mothers during one week in March 1946 in England, Scotland and Wales - it was not possible to follow the full sample due to funding constraints at that time [32]*.* The sample was socially stratified to include roughly equal numbers of children across all paternal occupational social class categories. This was achieved by including all those babies whose fathers worked in a non-manual or an agricultural occupation, and a random selection of one-in-four children whose fathers were employed in manual occupations. 672 children born to unmarried mothers were excluded, as it was assumed that they would be adopted at birth and would be too difficult to trace. 180 multiple births were also excluded, as were thought to be too small of a sample size for separate analyses. In line with the British population in 1946, there are very few participants from ethnic minority groups within the sample. At ages 60-64, the cohort were broadly similar to the national population of the same age on a range of sociodemographic indicators [32]. The 24^th^ follow-up occurred between 2014 and 2016, with a postal survey at age 68, and a home visit at 69 years of age, through which 2,638 participants (49%) provided information [33]. Prior loss to follow-up, up to age 69 was due to withdrawal (N = 633), emigration (N = 583), being untraceable (N = 432), and death (N = 995). A Strengthening the Reporting of Observational Studies in Epidemiology (STROBE) checklist is available in Table 1 in S1 File.

### Ethical approval, consent to participate and data access

For the latest data collection wave, ethical approval was given by the National Research Ethics Service Committee London Queen Square (14/LO/1073) and by the Scotland A Research Ethics Committee (14/SS/1009). Written informed consent was obtained from all individual participants included in NSHD for each wave of data collection. All participant data was anonymised for analysis and was first accessed by the lead author for research purposes on 26/01/2022.

### Measures

#### Air pollution.

Multiple air pollution models were utilised, relating to differing availability of national measurement data over time. Nitrogen dioxide (NO_2_) was estimated in 1991 (at cohort age 45 years) through land-use regression models [34]. In 2001 (at cohort age 55 years), exposure to NO_2_ and PM_10_ were estimated with the use of land-use regression modelling from Ruimte voor Geoinformatie [35]. Lastly, in 2010 (at cohort age 64), NO_2_, NO_x_, PM_10_, PM_2.5_, PM_coarse_ and PM_2.5_abs were estimated through European Study of Cohorts for Air Pollution Effects (ESCAPE) land-use regression models [36,37]. No subsequent air pollution estimates were available. All exposures are reported in µg/m^3^ (aside from PM_2.5_abs reported in 10^-5^m^-1^).

Each air pollution exposure (for years 1991, 2001 and 2010) was linked to the participants’ address for their age at the closest wave of data collection in this cohort (age 43 in 1989, 53 in 1999, and 60-64 in 2006-10). This reference age is used in further analyses and methods reporting. Model specifications varied between pollutants and time-points but generally improved across the assessment period. Full model details are provided in Table 2 in S1 File.

#### Psychological distress.

Psychological distress was assessed at ages 53, 60-64 and 69 years using the 28-item version of the General Health Questionnaire (GHQ-28), a self-reported measure of psychological distress [38]. The 28 items were scored on a 4-point Likert scale (with 0 considered as ‘low distress’ through to 3 indicating ‘high distress’) and responses were summed to create a total score with a range of 0 to 84, with higher scores representing higher psychological distress. The symptoms addressed in the scale cover elements of somatic symptomology, anxiety, social dysfunction and depression. The total scores were natural log-transformed for normality [39].

#### Covariates.

Covariates included were assigned sex at birth, social class at age 43 or 53, as defined by a condensed grouping of the UK Registrar General’s social class scheme [40] (“Professional” and “Intermediate”, “Skilled (non-manual)”, “Skilled (manual)”, and “Partly skilled” or “Unskilled”), neighbourhood deprivation at age 53 or 60 (defined by the percentage of people in the local area who are not employed) [41], and self-reported cigarette smoking status (“Never Smoked”, “Ex-Smoker”, “Smoker”) at age 43, 53 or 60-64.

To address confounding by previous exposure, we utilised additional indicators of pollutant exposure across the life course. At birth, the Douglas-Waller Index provided a rough marker of exposure through the use of coal consumption in participants’ county borough (regions defined by a population limit of 75,000), and was reported as “Low”, “Medium” or “High” [42]. At age 25, we included black smoke (BS) and SO_2_ exposures from Chronic Health Effects of Smoke and SO_2_ (CHESS) land-use regression models [43]. As CHESS models were available only up to age 43, had relatively poor statistical performance in year 1991, and as BS exposure has not been captured routinely beyond this point, we only included BS and SO_2_ as additional confounders for exposure earlier in life.

To control for reverse directionality by previous mental health problems, we included assessments from a teacher-rated survey conducted in childhood at ages 13-15, which was a precursor to the Rutter B2 teacher questionnaire [44], with total scores split into “Absent”, “Mild”, and “Severe” for internalising and externalising components, as previously described [45]. In adulthood, the Present State Examination (PSE), a semi-structured clinical interview assessing the frequency and severity of psychiatric symptoms, was administered to participants at age 36 [46]. It was included as a continuous measure of pre-existing mental health problems.

### Statistical analysis

**Fig. 1:**
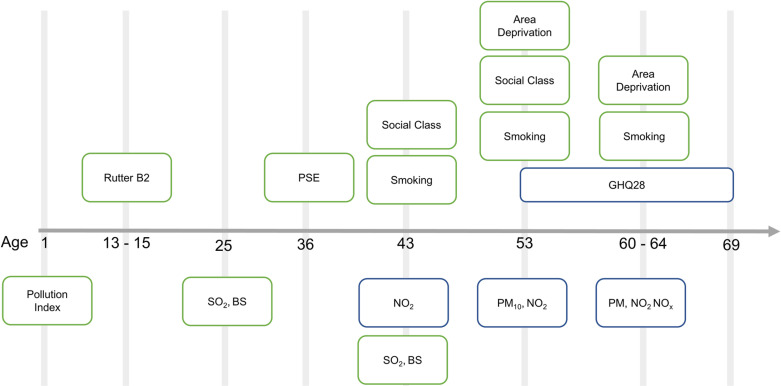
Timeline of measures used in this study. NO_2_ =  Nitrogen Dioxide, NO_x_ =  Nitrogen oxides, SO_2_ =  sulphur dioxide, BS =  black smoke, PM =  PM_10_, PM_2.5_, PM_coarse_, PM_2.5_abs. PM_10_ =  particulate matter 10µm or smaller, PM_2.5_ =  particulate matter 2.5µm or smaller, PM_coarse_ =  particulate matter of size 2.5µm-10µm, PM_2.5_abs =  absorption fraction related to particulate matter. PSE =  Present state examination. GHQ-28 =  General Health Questionnaire – 28. Blue =  main exposures and outcomes. Green =  covariates used.

All analyses were conducted in STATA 17.0 MP. A timeline of measures and a description of the models utilised are provided in Fig 1 and Table 3 in S1 File.

We compared participants who were lost to follow-up to those who were not between ages 43 and 69, by GHQ-28 completion. We examined differences in social class, deprivation, GHQ-28 total score, and NO_2_ exposure at time of drop-out. Differences between groups were examined by the χ^2^ test, t-test, or Mann-Whitney U test as appropriate.

All air pollution measures were re-scaled by the inter-quartile range (IQR), meaning associations with outcomes are represented in terms of one IQR increase in exposure. This allows for comparison of effects between pollutants which are on different concentration scales. All covariate measures were included from the closest available time-point to each exposure. Missing covariate data was addressed in age 60-64 exposures with age 69 GHQ-28 with the use of multiple imputation with chained equations (MICE) with 25 imputations. Marriage, childhood cognition and father’s social class were utilised as auxiliary variables.

Hypothesis 1: Long term exposures to elevated concentrations of pollutants would be associated with higher psychological distress over a 26-year (NO_2_), 16-year (PM_10_) and 5-year (NO_x_, PM_2.5,_ PM_coarse_ and PM_2.5_abs) period.

To examine the relationship over time between pollutant exposures and psychological distress for which we had multiple assessments, random intercept linear regression models using a maximum likelihood estimator were fitted for NO_2_ (at ages 43, 53 and 60-64) and PM_10_ (ages 53 and 60-64) with log-transformed GHQ-28 total score at ages 53, 60-64 and 69, with participant ID as the level 2 variable, and wave of data collection as the level 1 variable. Beta coefficients (β) and 95% confidence intervals (CIs) per IQR increase are reported. Fixed effects covariates are described below.

For pollutants available only at age 60-64 (NO_x,_ PM_2.5_, PM_coarse_ and PM_2.5_abs), associations were investigated separately between exposure to each air pollutant with log-transformed GHQ-28 total score at age 69 years using linear regression models. Beta coefficients (β) and 95% CIs were reported per IQR increase in exposure.

Both analysis methodologies were fitted as follows. Model 1 included participants with both psychological distress and the pollutant exposure available. Model 2 was adjusted for individual demographic factors of assigned sex at birth and social class (at age 43 or 53). Model 3 was further adjusted for smoking status at age of first exposure (age 43, 53 or 60-64). Model 4 included additionally neighbourhood factors of previous air pollution exposure (through the Douglas-Waller index at birth, and SO_2_ and BS exposure at age 25) and neighbourhood deprivation (at age 53 or 60). Model 5 further added previous mental health problems, at ages 13-15 and 36 (Rutter B2 precursor and the PSE, respectively).

Hypothesis 2: The association between air pollution exposure and psychological distress in mid-late adulthood would be stronger among those in lower socio-economic positions, utilising a proxy of SEP for participants in manual vs. non-manual jobs.

To test for potential effect modification by SEP, we first split social class into two categories of “Professional”, “Intermediate” and “Skilled (non-manual)” and compared to “Skilled (manual)”, “Partly skilled” and “Unskilled” [41]. We then repeated the above analyses with inclusion of an interaction term between each of the air pollutants and social class (manual vs non-manual) including in each of the models the same covariates as for hypothesis 1.

### Sensitivity analyses

Three sensitivity analyses were conducted. Firstly, co-pollutant models were included to assess confounding between pollutants as exposures are often highly correlated. For NO_2_, BS and SO_2_ were added at age 43 to NO_2_ models as there were no equivalent measures available for the whole period (age 43 to 60-64). For PM_10_, NO_2_ was included separately at age 53 and 60-64, and also at both time-points. For pollutants at age 60-64, gaseous and particulate pollutants were adjusted for each other (e.g., PM_2.5_ and NO_x_). We additionally report variance inflation factors for these full co-pollutant models to assess multicollinearity. Secondly, to index the effect of the highest levels of pollution exposure whilst ensuring parity of comparison and power, we conducted an extremes analysis for both models, which compared the association between the top quartile for exposure to each pollutant versus any other exposure quartile for that pollutant. Third, to assess whether exposure was related severity of outcome, a proxy of depression status (no vs. yes) was used instead of the continuous GHQ-28 score. This status was extrapolated from a combination of anti-depressant medication use at each time-point in this age range (ages 53, 60-64 and 69), combined with GHQ-28 scores above a threshold as previously constructed for this cohort [40]. Briefly, this proxy of depression was determined by a score of 5 or greater on the GHQ-28, utilising the original scoring method whereby scores of 2 and 3 on the Likert scale are recoded as 1, and scores of 0 and 1 are recoded as 0 and then item scores are summed [47]. Depression status was coded as 0 “No” (if the GHQ-28 total score was 4 or less and there was no reported use of anti-depressants) or 1 “Yes” (if the GHQ-28 total score was 5 or more, or there was reported use of anti-depressants, or both), independent of reports provided at previous time-points. The main analyses were then repeated using random intercept logistic regression models for NO_2_ and PM_10_, and logistic regression for NO_x,_ PM_2.5_, PM_coarse_ and PM_2.5_abs, reporting odds ratios (OR) and 95% CIs per IQR increase in exposure.

## Results

Sample characteristics are provided in Table 1. At age 69, there were 2125 participants with available GHQ-28 scores of which 51% were female (N = 1084). GHQ-28 scores decreased over time, from a median of 15 (IQR = 11, 20, N = 1970) at age 53, 14 (IQR = 11, 20, N = 1829) at age 60–64, and to 13 (IQR = 10, 18, N = 2125) at age 69. Air pollution exposures also decreased over time across the mid to older age assessment periods. For example, median NO_2_ exposure decreased from 29.3µg/m^3^ (IQR = 25.5, 35.1) at age 43, to 26.9µg/m^3^ (IQR = 24.2, 31.1) at age 53, and to 22.2µg/m^3^ (IQR = 17.6, 26.8) at age 60-64 (Table 1). Some exposures were highly intercorrelated, though this varied across time-points and models (e.g., at age 53, PM_10_ and NO_2_ had a correlation coefficient of 0.94, whilst at age 60-64, the correlation was 0.53) (Table 4 in S1 File).

**Table 1:** Descriptive statistics for air pollution exposures, psychological distress, and covariates for participants with GHQ-28 at age 69.

Variable	N (%)	Median	Interquartile Range	
**Assigned sex at birth – female**	1084			
**Exposures (µg/m** ^**3**^ **) – Age 43**				
** NO**_**2**_	1956	29.3	25.5, 35.1	
**Exposures (µg/m** ^**3**^ **) – Age 53**				
** NO**_**2**_	1947	26.9	24.2, 31.1	
** PM**_**10**_	1947	20.3	18.9, 21.8	
**Exposures (µg/m** ^**3**^ **) – Age 60-64**				
** NO**_**2**_	1948	22.2	17.6, 26.8	
** NO**_**x**_	1948	36.3	28.5, 44.6	
** PM**_**2.5**_	1799	9.6	8.9, 10.2	
** PM**_**10**_	1799	15.8	14.8, 16.7	
** PM**_**coarse**_	1799	6.1	5.8, 6.5	
** PM**_**2.5**_**abs (10**^**-5**^**m**^**-1**^)	1799	1.02	0.9, 1.2	
**Psychological Distress**				
** GHQ-28 - Age 53**	1970	15	11, 20	
** GHQ-28 - Age 60-64**	1829	14	11, 20	
** GHQ-28 - Age 69**	2125	13	10, 18	
**Depression proxy status**				
** Age 53**	416 (20.8)			
** Age 60-64**	414 (20.9)			
** Age 69**	416 (19.7)			
**Social class - age 43**				
** Professional or intermediate**	958 (49.9)			
** Non-manual skilled**	450 (23.4)			
** Manual skilled**	297 (15.5)			
** Partly skilled or unskilled**	216 (11.2)			
**Social class - age 53**				
** Professional or intermediate**	944 (48.9)			
** Non-manual skilled**	467 (24.2)			
** Manual skilled**	286 (14.8)			
** Partly skilled or unskilled**	232 (12.0)			
**Smoking status - age 43**				
** Current smoker**	493 (24.6)			
** Ex-smoker**	653 (32.5)			
** Never smoked**	862 (42.9)			
**Smoking status - age 53**				
** Current smoker**	379 (18.9)			
** Ex-smoker**	743 (37.0)			
** Never smoked**	889 (44.2)			
**Smoking status - age 60-64**				
** Current smoker**	192 (10.5)			
** Ex-smoker**	758 (41.4)			
** Never smoked**	882 (48.1)			
**Neighbourhood Deprivation**				
** Age 53**	2086	19.4	17.4, 21.4	
** Age 60**	1975	15.3	12.5, 17.9	
**Prior pollution exposure**				
** SO**_**2**_ **(µg/m**^**3**^**) - Age 25**	1991	67.8	53.1, 93.7	
** Black Smoke (µg/m** ^**3**^ **) - Age 25**	1991	35.1	24.2, 48.2	
** SO**_**2**_ **(µg/m**^**3**^**) - Age 43**	1956	27.8	24.0, 31.8	
** Black Smoke (µg/m** ^**3**^ **) - Age 43**	1956	10.9	8.7, 14.6	
**Pollution Index - Age 11**				
** Low**	347 (18.5)			
** 2**	629 (33.5)			
** 3**	501 (26.7)			
** High**	399 (21.3)			
**Internalising Problems – Age 13-15**				
** Absent**	1003 (52.7)			
** Mild**	719 (37.3)			
** Severe**	192 (10.1)			
**Externalising Problems – Age 13-15**				
** Absent**	1473 (77.3)			
** Mild**	323 (17.0)			
** Severe**	109 (5.7)			
**PSE – Age 36**	1927	1	0, 3	

PSE =  Present State Examination, BS = Black Smoke, SO_2_ =  Sulphur Dioxide, NO_2_ =  Nitrogen Dioxide, NO_X_ =  Nitrogen oxide, PM_10_ =  particulate matter size 10μm or smaller, PM_2.5_ =  particulate matter size 2.5μm or smaller, PM_coarse_ = Particulate matter size 2.5μm-10μm*,* PM_2.5_abs =  the particulate matter light absorption rate fraction. GHQ-28 =  General Health Questionnaire – 28*.*

There were no significant differences between air pollution exposure levels or GHQ-28 scores at age 53 in participants who were lost to follow-up between ages 53 and 69 and those who remained in the study. However, the group lost to follow-up were more likely to be from a manual or unskilled occupation or from areas of slightly higher deprivation (Table 5 in S1 File).

### Missing data

All covariates had missing data (aside from assigned sex at birth). Imputation resulted in a sample of N = 1946 for NO_x_ analyses, and N = 1797 for PM exposures, from N = 1176 and N = 1075 complete cases for model 5 respectively. Due to the extensive health and sociodemographic information available, missing at random data can be predicted by the observed data in this cohort [48].

Hypothesis 1: Long term exposures to elevated concentrations of pollutants would be associated with higher psychological distress over a 26-year (NO_2_), 16-year (PM_10_) and 5-year (NO_x_, PM_2.5,_ PM_coarse_ and PM_2.5_abs) period.

**Fig 2:**
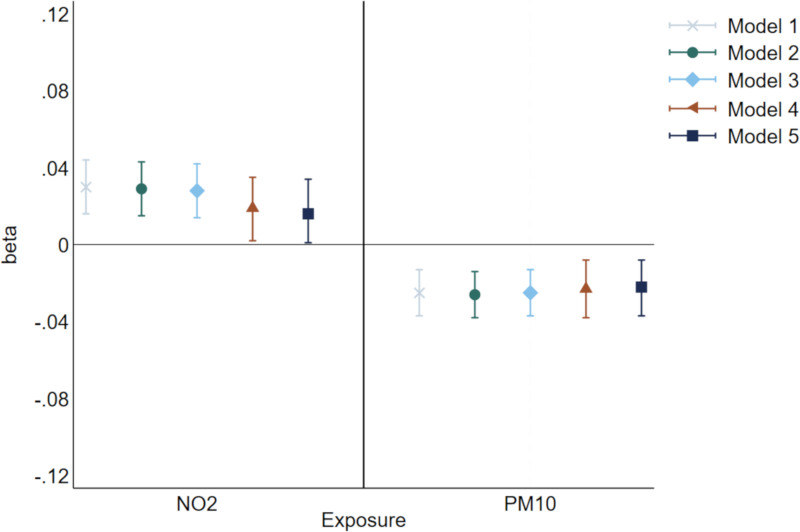
Graph of associations between exposure to rescaled, continuous NO_2_ and PM_10_ with continuous log-transformed GHQ-28 scores over a 26-year period for NO_2_ and 16 years for PM_10_ (between ages 43 to 69 years and 53 and 69 years, respectively). NO_2_ =  Nitrogen Dioxide, PM_10_ =  particulate matter 10µm or smaller. GHQ-28 =  General Health Questionnaire – 28. Crosses and lines represent β and 95% confidence intervals (95% CI) for Model 1, which is exposure and outcome only. Circles and lines represent β and 95% CI for Model 2, which is Model 1 +  assigned sex at birth and social class. Diamonds and lines represent β and 95% CI for Model 3, which is Model 2 +  cigarette smoking status. Triangles and lines represent β and 95% CI for Model 4, which is Model 3 +  neighbourhood deprivation and previous air pollution exposure. Squares and lines represent β and 95% CI for Model 5, which is Model 4 +  previous mental health problems. β and 95% CI represent the mean difference in log-transformed GHQ-28 score per interquartile range increase in exposure (μg/m^3^).

Longitudinal analyses were performed for NO_2_ (available at ages 43, 53 and 60-64) and PM_10_ (available at ages 53 and 60-64). Fully adjusted models showed evidence of positive associations between exposure to NO_2_ and log-transformed GHQ-28 scores over a 26-year period (β=0.023, 95%CI: 0.005, 0.040 per IQR increase of 8.01µg/m^3^) (Fig 2 and Table 2). For PM_10_ exposure and log-transformed GHQ-28 outcomes over a 16-year period, there was an inverse association between PM_10_ exposure and log-transformed GHQ-28 scores (β=-0.021, 95%CI: -0.037, -0.006 per IQR increase of 2.18µg/m^3^). For both pollutants we saw effect sizes reduce across models as we included more covariates, particularly with the inclusion of neighbourhood level covariates (Model 4 – deprivation and prior exposure). There was no evidence of an association between any of the air pollutant exposures (PM_2.5,_ NO_x_, PM_coarse_ and PM_2.5_abs) and continuous log-transformed GHQ-28 scores at age 69 in any model (e.g., PM_2.5_ exposure at age 60-64 with log-transformed GHQ-28 at age 69: β=0.002, 95%CI: -0.028, 0.032) (Table 3).

**Table 2:** Analysis of associations between NO_2_ and PM_10_ exposures with continuous log-transformed GHQ-28 scores over a 26-year period for NO_2_ and 16 years for PM_10_ (between ages 43 to 69 years and 53 and 69 years respectively).

EXPOSURE	N	MODEL 1	MODEL 2	MODEL 3	MODEL 4	MODEL 5
		β (95% Cl)	β (95% Cl)	β (95% Cl)	β (95% Cl)	β (95% Cl)
**NO**_**2**_	1497	**0.036** **(0.021, 0.051)**	**0.035** **(0.020, 0.050)**	**0.033** **(0.018, 0.048)**	**0.025** **(0.008, 0.043)**	**0.023** **(0.005, 0.040)**
**PM**_**10**_	1438	**-0.025** **(-0.038, -0.012)**	**-0.026** **(-0.039, -0.013)**	**-0.025** **(-0.038, -0.012)**	**-0.022** **(-0.038, -0.006)**	**-0.021** **(-0.037, -0.006)**

NO_2_ =  Nitrogen Dioxide, PM_10_ =  particulate matter size 10μm or smaller. GHQ-28 =  General Health Questionnaire – 28. p-value < 0.05 in bold and * . Model 1 =  exposure and outcome only. Model 2 =  Model 1 +  assigned sex at birth and social class. Model 3 =  Model 2 +  cigarette smoking status. Model 4 =  Model 3 +  neighbourhood deprivation and previous air pollution exposure. Model 5 =  Model 4 +  previous mental health problems. β and 95% confidence intervals (95% CI) represent the mean difference in log-transformed GHQ-28 score per interquartile range (μg/m^3^) increase in air pollutant levels.

**Table 3:** Analysis of between exposure to air pollution at age 60 – 64 (NO_x_, PM_2.5_, PM_coarse_, PM_2.5_abs) years with continuous log-transformed GHQ-28 scores at age 69.

EXPOSURE	N	MODEL 1	MODEL 2	MODEL 3	MODEL 4	MODEL 5
		β (95% Cl)	β (95% Cl)	β (95% Cl)	β (95% Cl)	β (95% Cl)
**NO**_**X**_	1946	0.017 (-0.007, 0.040)	0.014 (-0.009,0.038)	0.013 (-0.011, 0.036)	0.016 (-0.009, 0.041)	0.010 (-0.015, 0.034)
**PM**_**2.5**_	1797	0.015 (-0.014, 0.044)	0.008 (-0.020,0.037)	0.006 (-0.023, 0.034)	0.007 (-0.023, 0.038)	0.002 (-0.028, 0.032)
**PM**_**COARSE**_	1797	-0.014 (-0.031, 0.004)	-0.014 (-0.032,0.003)	-0.015 (-0.032, 0.003)	-0.015 (-0.032, 0.003)	-0.014 (-0.032, 0.003)
**PM**_**2.5**_ **ABS**	1797	-0.001 (-0.024, 0.021)	-0.004 (-0.026, 0.019)	-0.004 (-0.027, 0.018)	-0.005 (-0.030, 0.021)	-0.005 (-0.030, 0.020)

NO_X_ =  Nitrogen oxide, PM_2.5_ =  particulate matter size 2.5μm or smaller, PM_coarse_ = Particulate matter size 2.5μm-10μm, PM_2.5_abs =  the particulate matter light absorption rate fraction. GHQ-28 =  General Health Questionnaire – 28. Model 1 =  exposure and outcome only. Model 2 =  Model 1 +  assigned sex at birth and social class. Model 3 =  Model 2 +  cigarette smoking status. Model 4 =  Model 3 +  neighbourhood deprivation and previous air pollution exposure. Model 5 =  Model 4 +  previous mental health problems. β and 95% confidence intervals (95% CI) represent the mean difference in log-transformed GHQ-28 score per IQR increase in air pollutant exposure (μg/m^3^).

Hypothesis 2: The association between air pollution exposure and psychological distress in mid-late adulthood would be stronger among those in lower socio-economic positions, utilising a proxy of SEP for participants in manual vs. non-manual jobs.

We observed no evidence of effect modification by manual vs non-manual jobs in fully adjusted analyses (Tables 6 and 7 in S1 File).

### Sensitivity analyses

Firstly, in co-pollutant models, the main associations were maintained for NO_2_ (e.g., β=0.023, 95%CI: 0.006, 0.041, when controlled for BS at age 43) and for PM_10_ (controlled for NO_2_ at ages 53 and 60-64: β=-0.019, 95%CI: -0.035, -0.004) (Table 8 in S1 File). Interestingly, though not directly comparable to analysis that included all three time-points, when NO_2_ was included at age 53 and age 60-64 alongside PM_10_ it showed larger effect sizes than in single pollutant models (β=0.034, 95%CI: 0.015, 0.053). There was no evidence of association with log-transformed GHQ-28 scores when co-pollutants were included in fully adjusted models at age 60-64 (Table 9 in S1 File). In co-pollutant analyses, only PM_2.5_ and NO_x_ had variance inflation factors over 3, suggesting some degree of multicollinearity. Secondly, in extremes analyses, there was no evidence of robust associations for any of the pollutants and log-transformed GHQ-28 scores (Tables 10 and 11 in S1 File). The effect size for NO_2_ moved towards zero (β=0.011, 95%CI: -0.028, 0.050) and the beta for PM_10_ reversed and became positive (β=0.010, 95%CI: -0.030, 0.049), albeit the association was not statistically significant. Thirdly, there was no evidence of an association with depression status for any pollutant in fully adjusted models (Tables 12 and 13 in S1 File).

## Discussion

In this study we explored associations between air pollution exposure and psychological distress in middle to later adulthood, utilising repeated assessments in NO_2_ and PM_10_ and log-transformed GHQ-28. After controlling for previous air pollution exposure and mental health problems, and other individual and neighbourhood-level covariates, we found that exposure to higher levels of NO_2_ was associated with higher levels of psychological distress across this period. Conversely, we found that exposure to higher levels of PM_10_ was associated with lower psychological distress across a 16-year period. We found no associations with NO_x_, PM_2.5_, PM_coarse_ or PM_2.5_abs across a 5-year period and there was no evidence that the strength of any of the observed associations was modified by social class.

Our finding for NO_2_, while small, aligns with other evidence that suggests associations with higher exposure and increased mental health symptomology, particularly relating to depression, and with mixed results for psychological distress [8,13,14,25]. There have been a number of studies utilising repeated measures that found associations with NO_2_ and other pollutants, though these were not specific to older adults [10,13,14,25]. Utilising the US based Panel Study of Income Dynamics (PSID) (N = 6006), only census-area level unadjusted NO_2_ models found an association with psychological distress, as determined by the Kessler-6 scale [13]. In our models, we saw effect sizes reduce slightly with additional covariates, suggesting confounding of this association by a range of factors, particularly neighbourhood level effects and prior exposure. Further studies have utilised forms of the GHQ, mostly with a dichotomised outcome [14,25]. A large household study in the UK (N = 60,146) found increased odds of poorer mental health after higher exposure to NO_2_, SO_2_, PM_2.5_ and PM_10_ [14]. Focusing on older age, a study of older adults in Taiwan found that higher exposure to NO_2_ in the preceding year was associated with higher odds (OR = 1.209, 95%CI: 1.094, 1.336) of depression-like symptom [49]. Our results for NO_2_ (β=0.023, 95%CI: 0.005, 0.040 per IQR increase) reflect a small approximately 1.2% increase in GHQ-28 score, which may be important for improving psychological state on a population scale, particularly considering the GHQ is right-skewed in distribution. If casual, this may suggest that policy action on air pollution could help promote healthy ageing through reduced psychological distress. We could not find comparable studies for this age group specifically utilising a continuous outcome measure of psychological distress. Repeating studies such as this in older adults may be important as GHQ-28 reflects a broad range of symptoms beyond just depression, including anxiety [38].

We observed an unexpected counter-intuitive association of PM_10_ with GHQ-28 over time (i.e., higher exposure associated with lower psychological distress). This is contrary to other evidence that suggests no such association in older adults (e.g., OR = 0.992, 95%CI: 0.901, 1.093, for depressive symptoms in the same Taiwanese cohort) [49]. It could be that this is due to a lack of inclusion of neighbourhood level confounding, which was included in our study, or due to the much lower resolution exposure modelling (with 1 testing centre to each city or area). However, it is probable that this is a spurious result as there is no clear biological mechanism that could explain why higher levels of PM_10_ would be associated with lower psychological distress. This may be a result of exposure misclassification due to the low performance of the land-use regression model for PM_10_ at age 53 (r^2^ = 0.37) or due to multicollinearity (as the correlation coefficient of PM_10_ and NO_2_ was 0.94 at age 55 (Tables 2 and 4 in S1 File). The findings here, and in other cohorts, suggest that future work might be required on those particulate components most strongly correlated with NO_2_, more than components associated with coarser pollutants. This may imply differences in policy action as the primary source of NO_2_ in this context is traffic-related, compared to PM_10_ which may have industrial or other sources.

Many current studies and meta-analyses examining this association focus particularly on PM_2.5_ [8,13,19,20,49]. In the United States, an evaluation of 9 million older Medicaid users found significant increases in incidence of depression associated with higher exposure to PM_2.5_ (a 0.91% increase (95%CI: 0.02%, 1.81%) per 5μg/m^3^ increase in yearly exposure), over a 5-year moving mean, across 16 years of exposure [20]. Similarly, in the above all-age group PSID study they found associations with higher psychological distress after PM_2.5_ exposure (β=0.46; 95%CI: 0.35, 0.56 per 5µg/m^3^ increase) [13]. In our study, we found no significant association with PM_2.5_ (β=0.004, 95%CI: -0.026, 0.035, equivalent to an approximate 0.4% increase per IQR increase of 1.3µg/m^3^). As before, this could be due to residual confounding in PSID, with no neighbourhood level or prior exposure or outcomes considered in their models [13]. However, NSHD may also have insufficient power to detect an association if it exists. Though utilising binary outcomes, previous air pollution papers using the NSHD cohort and risk for mortality suggested a lack of power to detect the modest effect sizes seen in larger cohorts, and which may also be the case when examining depression status in our sensitivity analysis [50].

There may also be bias within the participants remaining in the study at this age range. Though exposure at age 43 was the same between participants lost to follow-up by age 53 and participants remaining in the study at age 69, the lost to follow-up group was more likely to have less skilled jobs and be living in areas of slightly higher deprivation. As lower SEP is associated with worse outcomes at age 69, the group lost to follow-up may have had worse outcomes [51] and this drop out could have limited our ability to identify associations between air pollution exposure and poorer mental health. We found no interaction effect between social class and air pollution exposure. There has been some investigation into the moderating influence of socio-economic factors, with mixed effects across different markers of SEP [10,20]. Qiu et al. found in a Medicaid sample of 8,907,422 participants in the US that risk of depression associated with increased exposure to PM_2.5_ was higher in Medicare eligible participants [20]. However, in the UK, Bakolis et al. found no effect modification by a latent class of socio-economic status [10]. The lack of interaction effects in our study could have been due to our occupational-based social class factors not fully capturing disparities seen through measures of individual SEP that capture other elements of social position such as income.

We did not explore mechanisms in this study though there are plausible candidates for explaining how NO_2_ may lead to the development of psychological distress, including through neuroinflammatory processes [21]. In animal models, NO_2_ has been shown to upregulate pro-inflammatory markers such as PGE_2_ which could lead to neurodegenerative processes and disruption and dysregulation of normal neural processes [52]. This may then lead to knock-on effects, where, for instance in early adolescence in humans, exposure to a range of pollutants, including NO_2_, have been found to disrupt functional connectivity implicated in a range of emotional disorders [53]. Other, indirect mediators such as physical activity changes have also been implicated in associations between air pollution and poorer mental health and may present alternative insights for developing interventions which aim to reduce individuals exposure to ambient pollution [25].

## Limitations

There are limitations to the methodology employed here. Residential exposure modelling may not reflect participants’ true exposure, particularly for participants in workplaces associated with high exposures [54]. Similarly, as our exposure modelling ends at age 60-64 it may be that exposure has changed for some participants up to age 69. However, we do not expect a difference in exposure within this period that could alter our findings, especially outside urban centres. Moreover, due to changes in air pollution monitoring in the UK since the 1940s and limitations in the monitoring data, it is not possible to directly estimate air pollution exposure to all pollutants over the life course with the same degree of certainty. The use of multiple models, as employed in these analyses, increases the risk of misclassification bias for each participant, as they employ different sources, resolutions, and modelling strategies. This may be a particular problem for the repeated measures for NO_2_ and PM_10_ which employ different exposure models across time and may result in varying correlations across time for these pollutants and with variable predictive performance. However, analysis between other methods of exposure modelling, such as back-extrapolation, show similar performance to the year-specific models included here [55]. Lastly, although the sample was selected at age 2 to represent all social classes and remains broadly similar over follow-up waves on some socio-demographic factors, there are limits to the generalisability of this cohort, due to a healthy cohort effect, exclusion of unmarried mothers and multiple births, and the population structure in England, Scotland and Wales at the time of initiation.

## Strengths

When considering these results in a wider life-course approach, this study represents a robust, longitudinal study, utilising extensive data available about participants, including repeated multiple measures of air pollution and psychological distress across the life course. With these factors, taken together with controls for previous exposure to air pollutants and prior mental health problems, we hope that this study can help direct policy and intervention appropriately (e.g., to reduce air pollution in areas near to older adults residence) [56] and support further research.

## Future research and recommendations

Future research using well-characterised longitudinal studies stemming from different countries, with a particular focus on low- and middle-income countries, should aim to replicate these findings and investigate potential mechanisms by employing standardised modelling methodologies across spatial and temporal scales.

## Conclusion

In this UK-based birth cohort, we examined longitudinal associations between exposure to a wide range of air pollutants and psychological distress between the ages of 43 and 69. We found associations between higher NO_2_ exposure in mid-late adulthood and higher psychological distress during this period after adjusting for a comprehensive range of individual and neighbourhood-level confounders, including prior mental health problems and pollution exposure. There was mixed evidence for associations between PM_10_ exposure and psychological distress during this period and no robust evidence of associations for other pollutants. Moreover, contrary to expectations, we found associations did not vary by social class. With populations that are ageing in many countries around the world, these findings suggest that policy action to reduce exposure to NO_2_ even in mid-life may support better mental health as individuals age.

## Supporting information

S1 File Supporting Information.
(DOCX)
