# Supplementary material for: Association of ambient air pollution exposure with psychological distress in mid and later adulthood: A 26-year prospective cohort study
Source: PLoS One. 2025 Mar 26;20(3):e0320332. doi: 10.1371/journal.pone.0320332 (PMC11940730; doi:10.1371/journal.pone.0320332)
Supplement: S1 File — (DOCX) [file pone.0320332.s001.docx]

S1 File Supporting Information

Canning et al., Association of ambient air pollution exposure with psychological distress in mid and later adulthood: a 26-year prospective cohort study

Contents

[Table 1: Strengthening the Reporting of Observational Studies in Epidemiology (STROBE) checklist 2](#_Toc189464648)

[Table 2: Model performance for air pollution exposure models. 7](#_Toc189464649)

[Table 3: Description of models used in analyses of the association between air pollution and mental health. 8](#_Toc189464650)

[Table 4: Correlation coefficients for air pollutants (NO_2_, PM_10_, NO_x_, PM_2.5_, PM_coarse_, PM_2.5_abs) at each exposure time point (age 43, 53 and 60-64) and between time points for NO_2_ and PM_10_. 10](#_Toc189464651)

[Table 5: Loss to follow-up at age 69 with key covariates and GHQ-28 scores at ages 53 and 69 respectively. 11](#_Toc189464652)

[Table 6: Effect modification analysis of associations between exposure to air pollution at age 60-64 (NO_x_, PM_2.5_, PM_coarse_, PM_2.5_abs) with continuous log-transformed GHQ-28 scores at age 69 by manual vs non-manual social class. 12](#_Toc189464653)

[Table 7: Effect modification analysis for associations of NO_2_ and PM_10_ with continuous log-transformed GHQ-28 scores over a 26-year period for NO_2_ and 16 years for PM_10_ (between ages 43 to 69 years and 53 and 69 years respectively) by manual vs non-manual social class. 13](#_Toc189464654)

[Table 8: Co-pollutant analysis for associations between NO_2_ and PM_10_ with continuous log-transformed GHQ-28 scores over a 26-year period for NO_2_ and 16 years for PM_10_ (between ages 43 to 69 years and 53 and 69 years respectively). 14](#_Toc189464655)

[Table 9: Co-pollutant analysis of associations between exposure to air pollution at age 60-64 (NO_x_, PM_2.5_, PM_coarse_, PM_2.5_abs) with continuous log-transformed GHQ-28 scores at age 69 in fully adjusted models (model 5) only. 15](#_Toc189464656)

[Table 10: Extremes analysis of associations between NO_2_ and PM_10_ with continuous log-transformed GHQ-28 scores over a 26-year period for NO_2_ and 16 years for PM_10_ (between ages 43 to 69 years and 53 and 69 years respectively). 16](#_Toc189464657)

[Table 11: Extremes analysis of associations between exposure to air pollution at age 60–64 (NO_x_, PM_2.5_, PM_coarse_, PM_2.5_abs) with continuous log-transformed GHQ-28 scores at age 69. 17](#_Toc189464658)

[Table 12: Associations between NO_2_ and PM_10_ with dichotomised proxy depression status over a 26-year period for NO_2_ and 16 years for PM_10_ (between ages 43 to 69 years and 53 and 69 years respectively). 18](#_Toc189464659)

[Table 13: Associations between exposure to air pollution at age 60-64 (NO_x_, PM_2.5_, PM_coarse_, PM_2.5_abs) with dichotomised proxy depression status at age 69. 19](#_Toc189464660)

# Table 1: Strengthening the Reporting of Observational Studies in Epidemiology (STROBE) checklist

|  | Item No | Recommendation | Included? |
| --- | --- | --- | --- |
| **Title and abstract** | 1 | (*a*) Indicate the study’s design with a commonly used term in the title or the abstract | Birth cohort and cohort study referenced in abstract and title |
|  |  | (*b*) Provide in the abstract an informative and balanced summary of what was done and what was found | Study details in abstract. |
| Introduction | | |  |
| Background/rationale | 2 | Explain the scientific background and rationale for the investigation being reported | Background and rationale provided in introduction for all hypotheses. |
| Objectives | 3 | State specific objectives, including any prespecified hypotheses | Specified hypotheses in final paragraph of introduction. |
| Methods | | |  |
| Study design | 4 | Present key elements of study design early in the paper | Cohort information provided in first paragraph of methods. |
| Setting | 5 | Describe the setting, locations, and relevant dates, including periods of recruitment, exposure, follow-up, and data collection | Cohort information provided in first, second and third paragraph of methods. |
| Participants | 6 | (*a*) Give the eligibility criteria, and the sources and methods of selection of participants. Describe methods of follow-up | Full reference to cohort papers provided for full detail. |
|  |  | (*b*) For matched studies, give matching criteria and number of exposed and unexposed | NA |
| Variables | 7 | Clearly define all outcomes, exposures, predictors, potential confounders, and effect modifiers. Give diagnostic criteria, if applicable | All variables defined in measures section of methods. |
| Data sources/ measurement | 8* | For each variable of interest, give sources of data and details of methods of assessment (measurement). Describe comparability of assessment methods if there is more than one group | All detail included for each variable, as well as reference for previous use if available. |
| Bias | 9 | Describe any efforts to address potential sources of bias | Examination of loss to follow-up explained in statistical analysis section. |
| Study size | 10 | Explain how the study size was arrived at | Available participant data broken down in results and methods. |
| Quantitative variables | 11 | Explain how quantitative variables were handled in the analyses. If applicable, describe which groupings were chosen and why | Each main variable has explanation on coding and transformations, with justification. |
| Statistical methods | 12 | (*a*) Describe all statistical methods, including those used to control for confounding | Included in statistical analyses section. |
|  |  | (*b*) Describe any methods used to examine subgroups and interactions | Interactions examined under hypothesis 2. |
|  |  | (*c*) Explain how missing data were addressed | Multiple imputation with chained equations was used for single time point analyses (NO_x_, PM_2.5,_ PM_coarse_PM2.5abs) as outlined in methods. Analysis for multiple time point pollutants (NO_2_ and PM_10_) analysed through mixed models utilising a maximum likelihood estimator. |
|  |  | (*d*) If applicable, explain how loss to follow-up was addressed | Bias from loss to follow-up addressed in cohort papers and in methods for this subsample. |
|  |  | (*e*) Describe any sensitivity analyses | Sensitivity analyses outlined in section “Sensitivity Analyses” in methods |
| Results | | |  |
| Participants | 13* | (a) Report numbers of individuals at each stage of study—eg numbers potentially eligible, examined for eligibility, confirmed eligible, included in the study, completing follow-up, and analysed | Reported in results section. |
|  |  | (b) Give reasons for non-participation at each stage | Provided in cohort papers referenced in methods. |
|  |  | (c) Consider use of a flow diagram | NA |
| Descriptive data | 14* | (a) Give characteristics of study participants (eg demographic, clinical, social) and information on exposures and potential confounders | See Table 1. |
|  |  | (b) Indicate number of participants with missing data for each variable of interest | To reference missing data a specific wave would have to be chosen. Full variables available in Table 1 and N included for the wave in which they were collected. |
|  |  | (c) Summarise follow-up time (eg, average and total amount) | Each data collection wave has follow-up time as per study design. |
| Outcome data | 15* | Report numbers of outcome events or summary measures over time | Reported GHQ-28 scores and depression status in Table 1. |
| Main results | 16 | (*a*) Give unadjusted estimates and, if applicable, confounder-adjusted estimates and their precision (eg, 95% confidence interval). Make clear which confounders were adjusted for and why they were included | Models outlined in methods section. Multiple tables provided for all results. |
|  |  | (*b*) Report category boundaries when continuous variables were categorized | NA |
|  |  | (*c*) If relevant, consider translating estimates of relative risk into absolute risk for a meaningful time period | NA – Coefficient for change reported as % increase in score in discussion. |
| Other analyses | 17 | Report other analyses done—eg analyses of subgroups and interactions, and sensitivity analyses | Sensitivity analyses reported on in text and in Supplementary tables. |
| Discussion | | |  |
| Key results | 18 | Summarise key results with reference to study objectives | Results outlined in terms of hypotheses in paragraph 1. |
| Limitations | 19 | Discuss limitations of the study, taking into account sources of potential bias or imprecision. Discuss both direction and magnitude of any potential bias | Limitations outlined in limitations section. |
| Interpretation | 20 | Give a cautious overall interpretation of results considering objectives, limitations, multiplicity of analyses, results from similar studies, and other relevant evidence | Results contextualised in existing evidence base. |
| Generalisability | 21 | Discuss the generalisability (external validity) of the study results | Reference to other studies, limitations and strengths of this study. |
| Other information | | |  |
| Funding | 22 | Give the source of funding and the role of the funders for the present study and, if applicable, for the original study on which the present article is based | Included after discussion. |

# Table 2: Model performance for air pollution exposure models.

| Year and Model | Air Pollution Variables | Model validation (r^2^) | Resolution |
| --- | --- | --- | --- |
| Age 25  CHESS |  |  |  |
|  | SO_2_ | 0.57 | 1000m x 1000m |
|  | BS | 0.41 | 1000m x 1000m |
| Age 45  CHESS |  |  |  |
|  | SO_2_ | 0.31 | 1000m x 1000m |
|  | BS | 0.34 | 1000m x 1000m |
|  |  |  |  |
| Age 45  Land use regression (NO_2_) |  |  |  |
|  | NO_2_ | 0.62 | 200m x 200m |
|  |  |  |  |
| Age 55 RGI |  |  |  |
|  | PM_10_ | 0.37 | 100m x 100m |
|  | NO_2_ | 0.61 | 100m x 100m |
|  |  |  |  |
| Age 60-64  ESCAPE |  |  |  |
|  | PM_2.5_ | 0.71 | 100m x 100m |
|  | PM_coarse_ | 0.56 | 100m x 100m |
|  | PM_2.5_abs | 0.92 | 100m x 100m |
|  | PM_10_ | 0.75 | 100m x 100m |
|  | NO_x_ | 0.78 | 100m x 100m |
|  | NO_2_ | 0.75 | 100m x 100m |

*SO_2_ = Sulphur Dioxide, BS = Black Smoke, NO_2_ = Nitrogen Dioxide, NO_x_ = Nitrogen oxides, PM_10_ = particulate matter 10µm or smaller, PM_2.5_ = particulate matter 2.5µm or smaller, PM_coarse_ = particulate matter of size 2.5µm-10µm, PM_2.5_abs= absorption fraction related to particulate matter. CHESS = Chronic Health Effects of Smoke and SO_2,_ ESCAPE = European Study of Cohorts for Air Pollution Effects, RGI = Ruimte voor Geoinformatie.*

# Table 3: Description of models used in analyses of the association between air pollution and mental health.

| Air Pollution Exposure | Mental Health Outcomes | Modelling framework |
| --- | --- | --- |
| ***Hypothesis 1: Long term exposures to elevated concentrations of pollutants would be associated with poorer mental health over a 26-year (NO_2_), 16-year (PM10) and 5-year (NO_x_, PM_2.5_, PM_coarse_ and PM_2.5_abs) period*** | | |
| NO_2_ at age 43, 53 and 60-64 | General Health Questionnaire-28 item (GHQ-28) at age 53, 60-64 and 69 | Random intercept linear regression models between all exposures and outcomes utilising repeated measures in individuals over time |
| PM_10_ at age 53 and 60-64 | General Health Questionnaire-28 item (GHQ-28) at age 60-64 and 69 | Random intercept linear regression models between all exposures and outcomes utilising repeated measures in individuals over time |
| NO_x_, PM_2.5_, PM_coarse_ and PM_2.5_abs, at age 60-64 | General Health Questionnaire-28 item (GHQ-28) at age 69 | Linear regression models with each exposure and each outcome at each time-point independently |
| ***Hypothesis 2: The association between air pollution exposure and poorer mental health in mid-late adulthood would be stronger among those in manual vs. non-manual roles.*** | | |
| NO_2_ at age 43, 53 and 60-64 | General Health Questionnaire-28 item (GHQ-28) at age 53, 60-64 and 69 | Random intercept linear regression models as above including interaction term between air pollution exposure and social class |
| PM_10_ at age 53 and 60-64 | General Health Questionnaire-28 item (GHQ-28) at age 60-64 and 69 | Random intercept linear regression models as above including interaction term between air pollution exposure and social class |
| NO_x_, PM_2.5_, PM_coarse_ and PM_2.5_abs, at age 60-64 | General Health Questionnaire-28 item (GHQ-28) at age 69 | Linear regression models with each exposure and each outcome independently including an interaction term between air pollution exposure and social class |

*NO_2_ = nitrogen dioxide, PM_10_ = particulate matter 10µm or smaller, NO_x_ = nitrogen oxide, PM_2.5_ = particulate matter 2.5µm or smaller, PM_coarse_ = particulate matter of size 2.5µm-10µm and* PM_2.5abs_ *= the absorption fraction related to particulate matter. GHQ-28 = General Health Questionnaire-28.*

# Table 4: Correlation coefficients for air pollutants (NO_2_, PM_10_, NO_x_, PM_2.5_, PM_coarse_, PM_2.5_abs) at each exposure time point (age 43, 53 and 60-64) and between time points for NO_2_ and PM_10_.

| Exposures | | | Correlation coefficient | | |
| --- | --- | --- | --- | --- | --- |
|  | NO_2_ (age 43) | NO_2_ (age 53) | NO_2_ (age 60-64) |  |  |
| NO_2_ (Age 43) | 1.00 |  |  |  |  |
| NO_2_ (Age 53) | 0.76 | 1.00 |  |  |  |
| NO_2_ (Age 60-64) | 0.64 | 0.72 | 1.00 |  |  |
|  | PM_10_ (age 53) | PM_10_ (age 60-64) |  |  |  |
| PM_10_ (Age 53) | 1.00 |  |  |  |  |
| PM_10_ (Age 60-64) | 0.34 | 1.00 |  |  |  |
| Age 53 |  |  |  |  |  |
|  | NO_2_ | PM_10_ |  |  |  |
| NO_2_ | 1.00 |  |  |  |  |
| PM_10_ | 0.94 | 1.00 |  |  |  |
| Age 60-64 |  |  |  |  |  |
|  | NO_2_ | NO_x_ | PM_10_ | PM_2.5_ | PM_coarse_ |
| NO_2_ | 1.00 |  |  |  |  |
| NO_x_ | 0.93 | 1.00 |  |  |  |
| PM_10_ | 0.53 | 0.54 | 1.00 |  |  |
| PM_2.5_ | 0.87 | 0.86 | 0.56 | 1.00 |  |
| PM_coarse_ | 0.71 | 0.64 | 0.59 | 0.58 | 1.00 |
| PM_2.5_abs | 0.16 | 0.20 | 0.75 | 0.16 | 0.38 |

*NO_2_ = nitrogen dioxide, PM_10_ = particulate matter 10µm or smaller, NO_x_ = nitrogen oxide, PM_2.5_ = particulate matter 2.5µm or smaller, PM_coarse_ = particulate matter of size 2.5µm-10µm and PM_2.5_abs = the absorption fraction related to particulate matter.*

# Table 5: Loss to follow-up at age 69 with key covariates and GHQ-28 scores at ages 53 and 69 respectively.

| Measure | Lost to follow-up at age 69? | | Statistical test result |
| --- | --- | --- | --- |
|  | No | Yes |  |
| GHQ-28 at Age 53 (N=2,902) (55.4%) (Median) | 15 | 15 | Z=-0.818, p = 0.413 |
| NO_2_ Exposure at Age 53 (N=2,670) (49.8%) |  |  |  |
| Quartile 1 | 449 (24.6) | 232 (27.4) | χ^2^=2.51, p = 0.474 |
| 2 | 461 (25.3) | 207 (24.4) |  |
| 3 | 475 (26.1) | 207 (24.4) |  |
| 4 | 438 (24.0) | 201 (23.7) |  |
| Social Class at Age 53 (N=2,710) (55.0%) |  |  |  |
| professional and intermediate | 925 (49.0) | 301 (36.6) | χ^2^=87.26, p < 0.001 |
| skilled (non-manual) | 459 (24.3) | 153 (18.6) |  |
| skilled (manual) | 279 (14.8) | 197 (23.9) |  |
| partly or unskilled | 224 (11.9) | 172 (20.9) |  |
| Deprivation at Age 53 (N=2,871) (54.8%) (Median) | 19.4 | 20.5 | t-test=-7.39, p < 0.001 |

*NO_2_ = Nitrogen Dioxide, GHQ-28 = General Health Questionnaire – 28. χ^2^ = chi-square* statistic

# Table 6: Effect modification analysis of associations between exposure to air pollution at age 60-64 (NO_x_, PM_2.5_, PM_coarse_, PM_2.5_abs) with continuous log-transformed GHQ-28 scores at age 69 by manual vs non-manual social class.

| Exposure | N | Model 1 | | Model 2 | | Model 3 | | Model 4 | | Model 5 | |
| --- | --- | --- | --- | --- | --- | --- | --- | --- | --- | --- | --- |
|  |  | β | 95% Cl | β | 95% Cl | β | 95% Cl | β | 95% Cl | β | 95% Cl |
| NO_x_ | 1946 | -0.010 | -0.068, 0.049 | -0.005 | -0.063, 0.054 | -0.009 | -0.067, 0.049 | -0.007 | -0.066, 0.052 | -0.011 | -0.069, 0.046 |
| PM_2.5_ | 1797 | -0.021 | -0.086, 0.045 | -0.013 | -0.078, 0.052 | -0.019 | -0.084, 0.046 | -0.018 | -0.083, 0.048 | -0.021 | -0.085, 0.044 |
| PM_coarse_ | 1797 | -0.005 | -0.045, 0.035 | -0.007 | -0.047, 0.033 | -0.006 | -0.046, 0.034 | -0.006 | -0.046, 0.035 | -0.008 | -0.047, 0.031 |
| PM_2.5_abs | 1797 | -0.006 | -0.058, 0.046 | -0.006 | -0.058, 0.045 | -0.009 | -0.062, 0.043 | -0.009 | -0.061, 0.044 | -0.012 | -0.063, 0.040 |

*NO_x_ = Nitrogen oxide, PM_2.5_= particulate matter size 2.5µm or smaller, PM_coarse_ = Particulate matter size 2.5µm-10µm, PM_2.5_abs = the particulate matter light absorption rate fraction. GHQ-28 = General Health Questionnaire – 28. p-value <0.05 in bold and *. Model 1 = outcome and exposure. Model 2 = Model 1 + assigned sex at birth. Model 3 = Model 2 + cigarette smoking, Model 4 = Model 3 + neighbourhood deprivation and previous air pollution exposure, Model 5 = Model 4 + previous mental health problems.* *β and 95% confidence intervals (95% CI) represent the effect of membership to manual social class grades on mean difference for GHQ-28 score per interquartile range (μg/m^3^) increase in air pollutant levels.*

# Table 7: Effect modification analysis for associations of NO_2_ and PM_10_ with continuous log-transformed GHQ-28 scores over a 26-year period for NO_2_ and 16 years for PM_10_ (between ages 43 to 69 years and 53 and 69 years respectively) by manual vs non-manual social class.

| Exposure | N | Model 1 | | Model 2 | | Model 3 | | Model 4 | | Model 5 | |
| --- | --- | --- | --- | --- | --- | --- | --- | --- | --- | --- | --- |
|  |  | β | 95% Cl | β | 95% Cl | β | 95% Cl | β | 95% Cl | β | 95% Cl |
| NO_2_ | 1497 | -0.005 | -0.037, 0.027 | -0.003 | -0.034, 0.029 | -0.003 | -0.035, 0.029 | -0.002 | -0.034, 0.030 | -0.002 | -0.033, 0.030 |
| PM_10_ | 1438 | 0.013 | -0.016, 0.042 | 0.016 | -0.013, 0.045 | 0.016 | -0.013, 0.045 | 0.015 | -0.014, 0.044 | 0.016 | -0.013, 0.045 |

*NO_2_ = Nitrogen Dioxide, PM_10_ = particulate matter size 10µm or smaller, GHQ-28 = General Health Questionnaire – 28. p-value <0.05 in bold and *. Model 1 = outcome and exposure. Model 2 = Model 1 + assigned sex at birth. Model 3 = Model 2 + cigarette smoking, Model 4 = Model 3 + neighbourhood deprivation and previous air pollution exposure, Model 5 = Model 4 + previous mental health problems.* *β and 95% confidence intervals (95% CI) represent the effect of membership to lower social class grades on mean difference for GHQ-28 score per interquartile range (μg/m^3^) increase in air pollution.*

# Table 8: Co-pollutant analysis for associations between NO_2_ and PM_10_ with continuous log-transformed GHQ-28 scores over a 26-year period for NO_2_ and 16 years for PM_10_ (between ages 43 to 69 years and 53 and 69 years respectively).

|  |  | |  |  |  |  |  |
| --- | --- | --- | --- | --- | --- | --- | --- |
| Co-Pollutant Exposure Age |  | Outcome  (GHQ-28) | | | |  | |
|  | N | β (95% CI) | | β (95% CI) | β (95% CI) | Variance inflation factor | |
|  |  | Main exposure | | Co-pollutant | | NO_2_ | Co-pollutant |
|  |  | **NO_2_** | | **BS** | **SO_2_** |  |  |
| Age 43 | 1442 | **0.023***  **(0.006, 0.041)** | | -0.008  (-0.040, 0.023) | - | 1.47 | 1.66 |
| Age 43 | 1442 | **0.024***  **(0.007, 0.042)** | | - | -0.022  (-0.053, 0.008) | 1.48 | 1.66 |
|  |  | **PM_10_** | | **NO_2_** | - |  |  |
| Age 53 | 1304 | **-0.025***  **(-0.043, -0.008)** | | 0.011  (-0.024, 0.045) | - | 1.72 | 2.17 |
| Age 60-64 | 1070 | **-0.026***  **(-0.043, -0.009)** | | 0.002  (-0.037, 0.040) | - | 1.52 | 1.59 |
| Age 53 and 60-64 | 1438 | **-0.019***  **(-0.035, -0.004)** | | **0.034***  **(0.015, 0.053)** | - | 1.33 | 1.45 |

*NO_2_ = Nitrogen Dioxide, PM_10_ = particulate matter size 10µm or smaller, GHQ-28 = General Health Questionnaire – 28. p-value <0.05 in bold and *. Each co-pollutant was added to fully adjusted models (Model 5) All results are fully adjusted models, including assigned sex at birth, social class, cigarette smoking, neighbourhood deprivation and previous air pollution exposure, previous mental health problems. β and 95% confidence intervals (95% CI) represent the mean difference in GHQ-28 score per interquartile range (μg/m^3^) increase in air pollutant levels*

# Table 9: Co-pollutant analysis of associations between exposure to air pollution at age 60-64 (NO_x_, PM_2.5_, PM_coarse_, PM_2.5_abs) with continuous log-transformed GHQ-28 scores at age 69 in fully adjusted models (model 5) only.

| Exposures modelled |  | GHQ-28 (Age 69) | | | |  | |
| --- | --- | --- | --- | --- | --- | --- | --- |
|  | N | β (95% CI) | β (95% CI) | β (95% CI) | β (95% CI) | Variance inflation factor | |
|  |  | NO_x_ | PM_2.5_ | PM_coarse_ | PM_2.5_abs | NO_x_ | Co-pollutant |
| PM_2.5_ + NO_x_ | 1797 | 0.006  (-0.039, 0.052) | -0.004  (-0.056, 0.049) | - | - | 3.40 | 3.48 |
| PM_coarse_ + NO_x_ | 1797 | 0.008  (-0.019, 0.034) | - | -0.015  (-0.033, 0.020) | - | 1.19 | 1.04 |
| PM_2.5_abs + NO_x_ | 1797 | 0.010  (-0.021, 0.041) | - | - | -0.010  (-0.040, 0.020) | 1.61 | 1.76 |

*NO_x_ = Nitrogen oxide, PM_2.5_= particulate matter size 2.5µm or smaller, PM_coarse_ = Particulate matter size 2.5µm-10µm, PM_2.5_abs = the particulate matter light absorption rate fraction. GHQ-28 = General Health Questionnaire – 28. p-value <0.05 in bold and *. All results are fully adjusted models, including assigned sex at birth, social class, cigarette smoking, neighbourhood deprivation and previous air pollution exposure, previous mental health problems. β and 95% confidence intervals (95% CI) represent the mean difference for GHQ-28 score per quartile (μg/m^3^) increase in air pollutant levels.*

# Table 10: Extremes analysis of associations between NO_2_ and PM_10_ with continuous log-transformed GHQ-28 scores over a 26-year period for NO_2_ and 16 years for PM_10_ (between ages 43 to 69 years and 53 and 69 years respectively).

| Exposure | N | Model 1 | | Model 2 | | Model 3 | | Model 4 | | Model 5 | |
| --- | --- | --- | --- | --- | --- | --- | --- | --- | --- | --- | --- |
|  |  | β | 95% Cl | β | 95% Cl | β | 95% Cl | β | 95% Cl | β | 95% Cl |
| NO_2_ | 1497 | 0.018 | -0.019, 0.054 | 0.016 | -0.020, 0.052 | 0.015 | -0.020, 0.051 | 0.015 | -0.024, 0.055 | 0.011 | -0.028, 0.050 |
| PM_10_ | 1438 | 0.014 | -0.025, 0.053 | 0.012 | -0.027, 0.050 | 0.011 | -0.028, 0.050 | 0.008 | -0.032, 0.048 | 0.010 | -0.030, 0.049 |

*NO_2_ = Nitrogen Dioxide, PM_10_ = particulate matter size 10µm or smaller, GHQ-28 = General Health Questionnaire – 28. p-value <0.05 in bold and *. Model 1 = outcome and exposure. Model 2 = Model 1 + assigned sex at birth and social class. Model 3 = Model 2 + cigarette smoking, Model 4 = Model 3 + neighbourhood deprivation and previous air pollution exposure, Model 5 = Model 4 + previous mental health problems. β and 95% confidence intervals (95% CI) represent the mean difference in log-transformed GHQ-28 score between the top quartile and 3 lower quartiles of exposure (μg/m^3^) in air pollutant levels.*

# Table 11: Extremes analysis of associations between exposure to air pollution at age 60–64 (NO_x_, PM_2.5_, PM_coarse_, PM_2.5_abs) with continuous log-transformed GHQ-28 scores at age 69.

| Exposure | N | Model 1 | | Model 2 | | Model 3 | | Model 4 | | Model 5 | |
| --- | --- | --- | --- | --- | --- | --- | --- | --- | --- | --- | --- |
|  |  | β | 95% Cl | β | 95% Cl | β | 95% Cl | β | 95% Cl | β | 95% Cl |
| NO_x_ | 1946 | 0.007 | -0.039, 0.054 | 0.004 | -0.042, 0.050 | 0.001 | -0.045, 0.047 | 0.003 | -0.046, 0.052 | -0.009 | -0.057, 0.039 |
| PM_2.5_ | 1797 | -0.002 | -0.051, 0.047 | -0.011 | -0.059, 0.038 | -0.015 | -0.063, 0.034 | -0.015 | -0.066, 0.036 | -0.019 | -0.068, 0.031 |
| PM_coarse_ | 1797 | -0.020 | -0.069, 0.028 | -0.021 | -0.069, 0.027 | -0.022 | -0.070, 0.026 | -0.022 | -0.070, 0.027 | -0.028 | -0.075, 0.020 |
| PM_2.5_abs | 1797 | -0.016 | -0.065, 0.033 | -0.016 | -0.064, 0.033 | -0.017 | -0.065, 0.031 | -0.019 | -0.073, 0.035 | -0.021 | -0.074, 0.032 |

*NO_x_ = Nitrogen oxide, PM_2.5_= particulate matter size 2.5µm or smaller, PM_coarse_ = Particulate matter size 2.5µm-10µm, PM_2.5_abs = the particulate matter light absorption rate fraction. GHQ-28 = General Health Questionnaire – 28. p-value <0.05 in bold and *. Model 1 = outcome and exposure. Model 2 = Model 1 + assigned sex at birth and social class. Model 3 = Model 2 + cigarette smoking, Model 4 = Model 3 + neighbourhood deprivation and previous air pollution exposure, Model 5 = Model 4 + previous mental health problems.* *β and 95% confidence intervals (95% CI) represent the mean difference in log-transformed GHQ-28 score between the top quartile and 3 lower quartiles of exposure (μg/m^3^).*

# Table 12: Associations between NO_2_ and PM_10_ with dichotomised proxy depression status over a 26-year period for NO_2_ and 16 years for PM_10_ (between ages 43 to 69 years and 53 and 69 years respectively).

| Exposure | N | Model 1 | | Model 2 | | Model 3 | | Model 4 | | Model 5 | |
| --- | --- | --- | --- | --- | --- | --- | --- | --- | --- | --- | --- |
|  |  | OR | 95% Cl | OR | 95% Cl | OR | 95% Cl | OR | 95% Cl | OR | 95% Cl |
| NO_2_ | 1497 | 1.164 | 1.024, 1.323 | 1.155 | 1.017, 1.312 | 1.137 | 1.001, 1.292 | 1.132 | 0.972, 1.319 | 1.105 | 0.950, 1.287 |
| PM_10_ | 1438 | 0.906 | 0.791, 1.038 | 0.897 | 0.784, 1.027 | 0.917 | 0.803, 1.048 | 0.889 | 0.760, 1.039 | 0.889 | 0.761, 1.038 |

*NO_2_ = Nitrogen Dioxide, PM_10_ = particulate matter size 10µm or smaller, GHQ-28 = General Health Questionnaire – 28. p-value <0.05 in bold and *. Model 1 = outcome and exposure. Model 2 = Model 1 + assigned sex at birth and social class. Model 3 = Model 2 + cigarette smoking, Model 4 = Model 3 + neighbourhood deprivation and previous air pollution exposure, Model 5 = Model 4 + previous mental health problems. Odds ratios (OR) and 95% confidence intervals (95% CI) represent the increase of risk of depression status per interquartile range (μg/m^3^) increase in air pollutant levels.*

# Table 13: Associations between exposure to air pollution at age 60-64 (NO_x_, PM_2.5_, PM_coarse_, PM_2.5_abs) with dichotomised proxy depression status at age 69.

| Exposure | N | Model 1 | | Model 2 | | Model 3 | | Model 4 | | Model 5 | |
| --- | --- | --- | --- | --- | --- | --- | --- | --- | --- | --- | --- |
|  |  | OR | 95% Cl | OR | 95% Cl | OR | 95% Cl | OR | 95% Cl | OR | 95% Cl |
| NO_x_ | 1946 | 1.116 | 0.985, 1.265 | 1.110 | 0.977, 1.260 | 1.098 | 0.966, 1.248 | 1.111 | 0.967, 1.276 | 1.086 | 0.942, 1.251 |
| PM_2.5_ | 1797 | 1.175 | 1.006, 1.372 | 1.144 | 0.979, 1.338 | 1.132 | 0.967, 1.324 | 1.140 | 0.964, 1.349 | 1.120 | 0.943, 1.330 |
| PM_coarse_ | 1797 | 0.974 | 0.882, 1.075 | 0.973 | 0.880, 1.075 | 0.970 | 0.877, 1.073 | 0.972 | 0.877, 1.076 | 0.970 | 0.873, 1.077 |
| PM_2.5_abs | 1797 | 1.002 | 0.884, 1.136 | 0.992 | 0.875, 1.125 | 0.988 | 0.871, 1.121 | 0.988 | 0.855, 1.141 | 0.985 | 0.849, 1.143 |

*NO_x_ = Nitrogen oxide, PM_2.5_= particulate matter size 2.5µm or smaller, PM_coarse_ = Particulate matter size 2.5µm-10µm, PM_2.5_abs = the particulate matter light absorption rate fraction. GHQ-28 = General Health Questionnaire – 28. P-value <0.05 in bold and *. Model 1 = outcome and exposure. Model 2 = Model 1 + assigned sex at birth and social class. Model 3 = Model 2 + cigarette smoking, Model 4 = Model 3 + neighbourhood deprivation and previous air pollution exposure, Model 5 = Model 4 + previous mental health problems. Odds ratios (OR) and 95% confidence intervals (95% CI) represent the increase of risk of depression status per interquartile range (μg/m^3^) increase in air pollutant levels.*
